# Playing with Opening and Closing of Heterocycles: Using the Cusmano-Ruccia Reaction to Develop a Novel Class of Oxadiazolothiazinones, Active as Calcium Channel Modulators and P-Glycoprotein Inhibitors

**DOI:** 10.3390/molecules191016543

**Published:** 2014-10-14

**Authors:** Domenico Spinelli, Roberta Budriesi, Barbara Cosimelli, Elda Severi, Matteo Micucci, Massimo Baroni, Fabio Fusi, Pierfranco Ioan, Simon Cross, Maria Frosini, Simona Saponara, Rosanna Matucci, Camillo Rosano, Maurizio Viale, Alberto Chiarini, Emanuele Carosati

**Affiliations:** 1 Dipartimento di Chimica “G. Ciamician”, Alma Mater Studiorum-Università di Bologna, Via F. Selmi 2, Bologna 40126, Italy; E-Mail: domenico.spinelli@unibo.it; 2 Dipartimento di Farmacia e Biotecnologie, Alma Mater Studiorum-Università di Bologna, Via Belmeloro 6, Bologna 40126, Italy; E-Mails: matteo.micucci2@unibo.it (M.M.); pierfranco.ioan3@unibo.it (P.I.); alberto.chiarini@unibo.it (A.C.); 3 Dipartimento di Farmacia, Università di Napoli “Federico II”, Via D. Montesano 49, Napoli 80131, Italy; E-Mails: barbara.cosimelli@unina.it (B.C.); elda.severi@unina.it (E.S.); 4 Molecular Discovery Ltd., 215 Marsh Road, Pinner, Middlesex HA5 5NE, UK; E-Mails: massimo@moldiscovery.com (M.B.); simon@moldiscovery.com (S.C.); 5 Dipartimento di Scienze della Vita, Università degli Studi di Siena, Via A. Moro 2, Siena 53100, Italy; E-Mails: fabio.fusi@unisi.it (F.F.); maria.frosini@unisi.it (M.F.); simona.saponara@unisi.it (S.S.); 6 Dipartimento di Neuroscienze, Area del Farmaco e Salute del Bambino (NEUROFARBA) Viale Pieraccini 6, Firenze 50139, Italy; E-Mail: rosanna.matucci@unifi.it; 7 IRCCS Azienda Ospedaliera Universitaria San Martino—IST Istituto Nazionale per la Ricerca sul Cancro, U.O.S. Biopolimeri e Proteomica, L.go R. Benzi, 10, Genova 16132, Italy; E-Mail: camillo.rosano@istge.it; 8 IRCCS Azienda Ospedaliera Universitaria San Martino—IST Istituto Nazionale per la Ricerca sul Cancro, U.O.C. Bioterapie, L.go R. Benzi, 10, Genova 16132, Italy; E-Mail: maurizio.viale@istge.it; 9 Dipartimento di Chimica, Biologia e Biotecnologie, Università di Perugia, Via Elce di Sotto 10, Perugia 06123, Italy

**Keywords:** 3D-QSAR, docking, Fingerprints for Ligands and Proteins (FLAP), L-Type Calcium Channels (LTCC), multidrug resistance (MDR1), negative inotropic activity, oxadiazolothiazinones, pharmacophore modeling, ternary complex

## Abstract

As a result of the ring-into-ring conversion of nitrosoimidazole derivatives, we obtained a molecular scaffold that, when properly decorated, is able to decrease inotropy by blocking L-type calcium channels. Previously, we used this scaffold to develop a quantitative structure-activity relationship (QSAR) model, and we used the most potent oxadiazolothiazinone as a template for ligand-based virtual screening. Here, we enlarge the diversity of chemical decorations, present the synthesis and* in vitro* data for 11 new derivatives, and develop a new 3D-QSAR model with recent *in silico* techniques. We observed a key role played by the oxadiazolone moiety: given the presence of positively charged calcium ions in the transmembrane channel protein, we hypothesize the formation of a ternary complex between the oxadiazolothiazinone, the Ca^2+^ ion and the protein. We have supported this hypothesis by means of pharmacophore generation and through the docking of the pharmacophore into a homology model of the protein. We also studied with docking experiments the interaction with a homology model of P-glycoprotein, which is inhibited by this series of molecules, and provided further evidence toward the relevance of this scaffold in biological interactions.

## 1. Introduction

Ring-into-ring conversions are basically ring transformations of heterocycles and constitute an interesting area for mechanistic studies and synthetic design [1,2,3,4]. The presence of heteroatom(s) favors the opening process, which yields the creation of new interesting functional groups that may even react to form new heterocycles [5,6]. Given the wide applicability domain of heterocyclic molecules, ranging from pharmaceuticals to herbicides, from veterinary to material chemistry [7], they have been jokingly named “jewelry rings studded with precious stones”, with the role of the precious stones being played by the heteroatoms [7].

The presence of heteroatom(s) in the ring causes a lowering of the resonance stabilization energy (particularly significant in five-membered rings), and introduces electronegativity differences between atoms of the ring [8,9]. These factors play a role in the ring-opening process of the carbon-heteroatom or heteroatom-heteroatom bonds, thus affecting the course and the final products of the reaction.

In this paper, we focus on some reactions of heterocycles, with special attention on the ring-into-ring conversion of nitrosoimidazole derivatives. One of these reactions led to a class of molecules, the oxadiazolo[3,4-*c*][1,4]thiazin-3-ones [10], biologically active against L-type calcium channels (LTCC) [11,12], and P-glycoprotein 1 (P-gp, or ATP-binding cassette sub-family B member 1, abbreviated as ABCB1) [13,14].

**Scheme 1:**
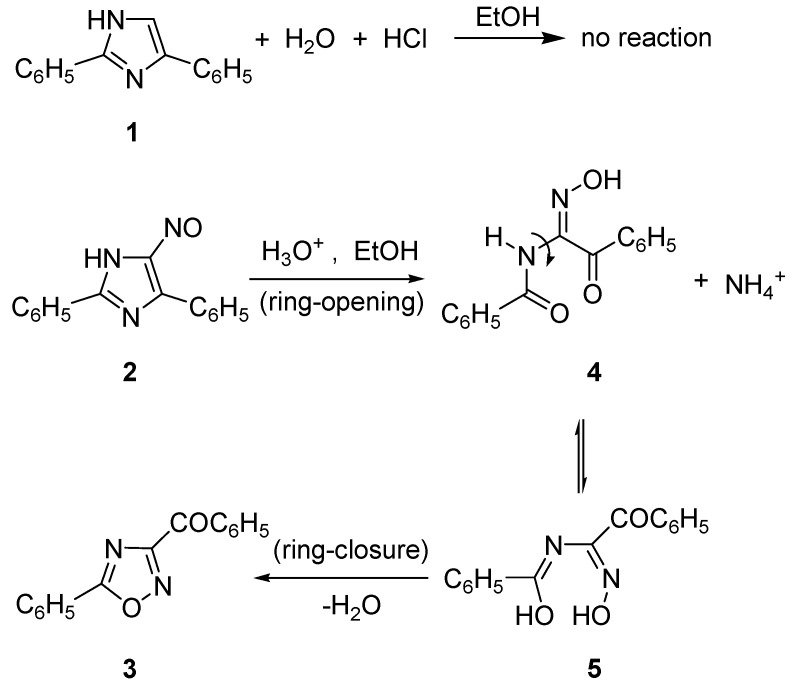
Difference between imidazole and nitrosoimidazole; the latter reacts with hydrochloric acid according to the “Cusmano-Ruccia reaction” mechanism.

The synthetic design of oxadiazolothiazinones began by observing the difference between 2,5-diphenylimidazole (**1**), stable to acids in ethanol at room temperature as well as at reflux, and 4-nitroso-2,5-diphenylimidazole (**2**), that reacts with mineral acids at room temperature (Scheme 1) [15,16]. The ring-opening/ring-closing reaction, that gives 3-benzoyl-5-phenyl-1,2,4-oxadiazole (**3**), was named as the “Cusmano-Ruccia reaction” [17].

**Scheme 2:**
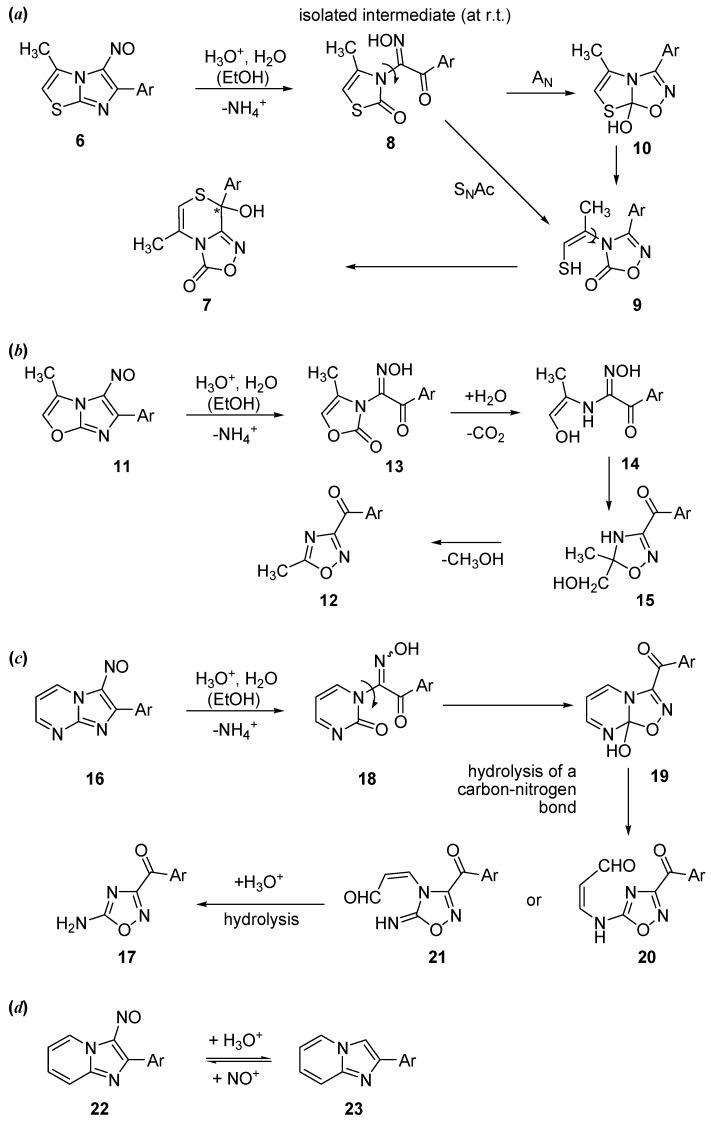
Mechanisms for different nitrosoimidazoles, condensed with thiazole (**a**), oxazole (**b**), pyrimidine (**c**) and pyridine (**d**). *Ar* stands for *para*-chlorophenyl.

By extending the study of the reactivity to condensed systems, we observed that 5-nitroso- imidazo[2,1-*b*][1,3]thiazoles also react easily with hydrochloric acid [10,18,19,20]. Thus, 3-methyl-5-nitroso-6-(4-chlorophenyl)imidazo[2,1-*b*][1,3]thiazole (**6**) (Scheme 2a) gives 5-methyl-8-chloro-phenyl-8-hydroxy-8*H*-[1,2,4]oxadiazolo[3,4-*c*][1,4]thiazin-3-one (**7**) [10,18].

We have also investigated the reactivity of 6-(4-chlorophenyl)-3-methyl-5-nitrosoimidazo[2,1-*b*][1,3]oxazole (**11**) [21], which gives the 3-(4-chlorobenzoyl)-5-methyl-1,2,4-oxadiazole (**12**) (Scheme 2b). In the case of imidazopyrimidines, the reaction leads to the 4-amino-1,2,4-oxadiazoles **17** (Scheme 2c) [22]; in contrast, a simple denitrosation occurs in the case of the imidazopyridines (Scheme 2d) [22].

By extending the abovementioned Cusmano-Ruccia reaction to several 5-nitroso-6-arylimidazo[2,1-*b*][1,3]thiazoles [19,20], we obtained a library of [1,2,4]oxadiazolo[3,4-*c*][1,4]thiazinones. Given the resemblance to diltiazem (**24**) [23], a well-known calcium channel blocker that has important therapeutic applications (angina pectoris, supraventricular arrhythmias, and mild to moderate systemic hypertension) [24,25], we hypothesized for these molecules a similar biological effect; thus, we investigated this experimentally, with functional assays, electrophysiology and binding experiments.

**Figure 1:**
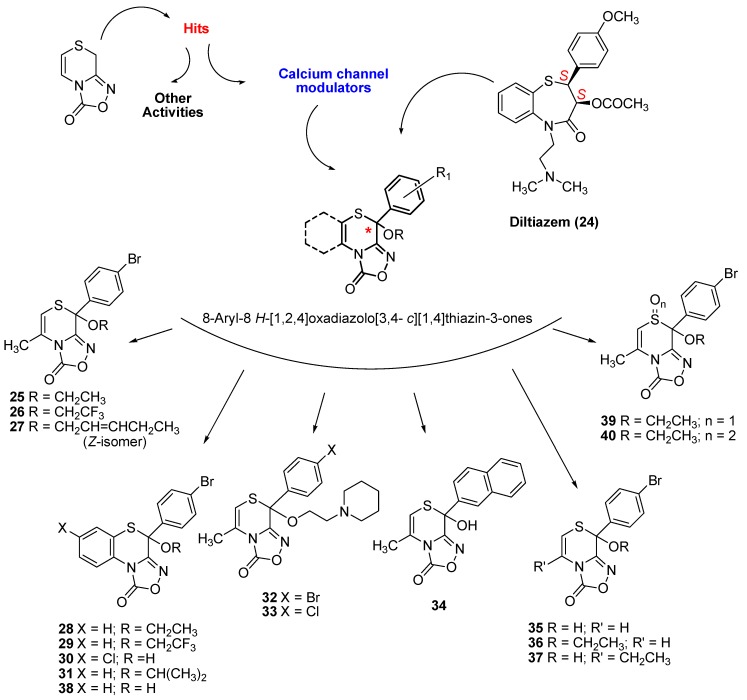
Structure of diltiazem (**24**), of published (compounds **25**–**29**, **38**) and novel oxadiazolothiazinone derivatives (compounds **30**–**37**, **39**, **40**), whose LTCC data is presented below.

Overall, experimental data indicated an activity to decrease the heart force (negative inotropic activity) comparable to that of diltiazem [11], but we achieved a compound 24-fold more potent than diltiazem (**25** in Figure 1) [12]. The negative inotropic activity was modulated by modifying the structure at the phenyl ring (at C8), at the C5-C6 bond, and by substituting the hydroxyl group with alkoxyl chains [11,12]. Recently, we published three other potent derivatives [26], characterized by the condensed benzothiazino ring and the CF_3_/CH_3_ substitution at the R chain (**26**–**28** in Figure 1). Here, we propose a further extension of the series of oxadiazolothiazinones to other peculiar molecules.

## 2. Results and Discussion

### 2.1. Synthesis of Oxadiazolothiazinones

*In vitro* data for 11 molecules (Figure 1) and the synthesis of most of them is presented here for the first time: two benzofused derivatives **30** and **31**, two derivatives **32** and **33** with a basic group at the alkyloxyl chain, one derivative **34** with a naphthyl group replacing the phenyl at C8, and another three (compounds **35**–**37**) with minor changes at the C5–C6 bond. The synthesis of one benzofused derivative **38** as well as of derivatives **39**, **40** with an oxidized sulphur atom of the thiazino moiety, has already been published [26,27].

**Scheme 3:**
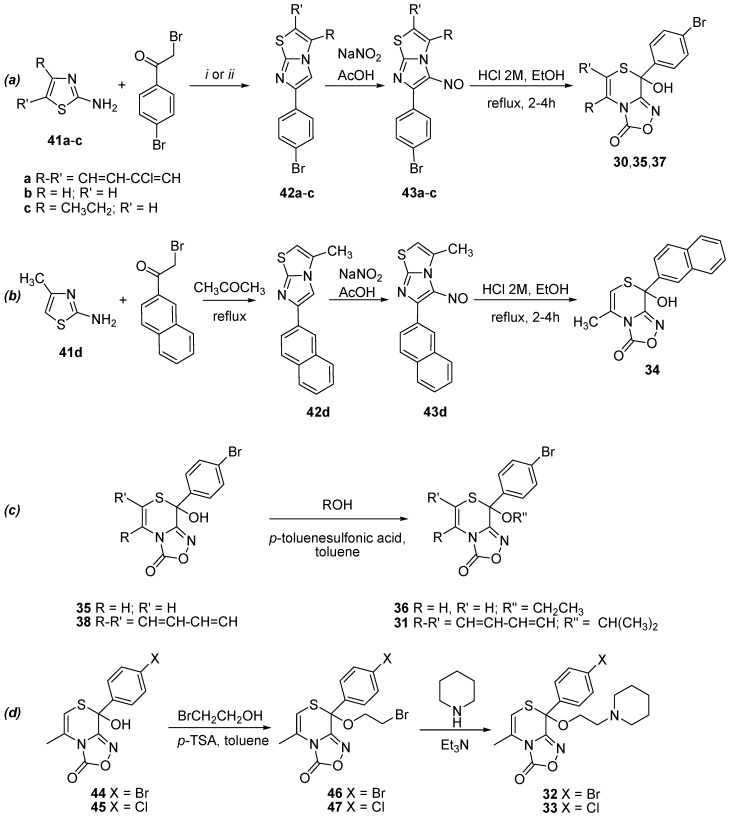
Synthetic route for hemithioacetals **30**, **35**, **37** starting from the opportune thiazole **41a**–**c** (**a**), synthetic route for hemithioacetal **34** starting from the aminothiazole **41d** (**b**), synthesis of thioketals **31** and **36** (**c**) and synthetic route for thioketals **32** and **33**, via the thioketals **46** and **47** respectively (**d**).

We obtained the new hemithioacetals **30**, **35**, and **37** following the general three-steps procedure of Scheme 3a: first, the treatment of 2-aminothiazoles **41a**–**c** with 2,4'-dibromoacetophenone gives the 4-bromophenylimidazo[2,1-*b*][1,3]thiazoles **42a**–**c**; these compounds, by reaction with sodium nitrite in acetic acid, give the nitroso derivatives **43a**–**c**; which in turn were converted into the corresponding [1,2,4]oxadiazol[3,4-*c*][1,4]thiazinones by the action of hydrochloric acid. An analogous route gives the new hemithioacetal **34** via compounds **42d** and **43d** (Scheme 3b). 

*Reagents and conditions*: (i) in the instance of **41a**: EtOH, Et_3_N, reflux; (ii) in the instance of **41b**, **c**: CH_3_COCH_3_, or CHCl_3_, reflux then HCl 2 M, EtOH, reflux.

The thioketals **31** and **36** were obtained by reacting **38** or **35** with the appropriate alcohol and refluxing in toluene in the presence of *p*-toluenesulfonic acid as catalyst (Scheme 3c). Two steps were necessary for the syntheses of **32** and **33**, obtained by reaction of **44** [19] or **45** [18] with 2-bromoethanol by refluxing in toluene in the presence of *p*-toluenesulfonic acid as catalyst (Scheme 3d) to give the corresponding intermediate **46** or **47**. These were subsequently reacted with piperidine to give **32** or **33**.

### 2.2. Biological Activity of Oxadiazolothiazinones as L-Type Calcium Channel Blockers

We assessed the cardiac activity of these oxadiazolothiazinones on guinea-pig left and right atria, as well as their relaxant activity on guinea-pig vascular (aorta) and nonvascular (ileum) smooth muscle. For some of them, with electrophysiology experiments we tested the modulation of the LTCC, whereas with binding experiments we tested diltiazem displacement from its binding site on LTCC.

#### 2.2.1. Functional Data

As with the reference compound **25**, all of the tested compounds were shown to be negative inotropic agents: half of them are more potent than diltiazem, and two of them are of the same order of potency as **25** (Table 1). Previous findings about the substituted phenyl ring (*p*-bromine more potent than *p*-chlorine) were confirmed: **35** resulted 32-fold more potent than its chlorine-analogue [11]. Concerning the substitution at C8 with an OH/OR group, compound **38** is much less potent than its ethoxy derivative **28** [26]. Pairwise comparisons also show that the methyl on the thiazino ring is not essential, but methyl/ethyl replacement is detrimental: **36** and **25** have comparable potency, whereas **37** is about 8-fold less potent than **35**. Relevant information arises from peculiar structural modifications: the effect of sulphur oxidation is detrimental for the potency, with the sulphone **40** slightly better than sulphoxide **39**; instead, the presence of chlorine at the fused benzothiazino ring enhances the potency. For all but two compounds (**32** and **33**) the negative chronotropic activity is absent. The weak chronotropic activity observed for **32** and **33** comes together with the reduction of the inotropic activity. The lack of selectivity observed for **32** and **33** is unique among the large series of oxadiazolothiazinone analogues that we have studied so far. In particular, the comparison with the data of the analogue with the CH_2_-cyclohexyl as lateral chain (compounds **20a**, **b** of reference [12], that are very selective toward inotropy), let us suppose that because of the piperidine ring the molecule interacts with different calcium channel isoforms, thus causing both weak inotropy and weak chronotropy.

The pharmacological profile of all compounds was extended to relaxant activities (Table 2), by using 80 mM K^+^-depolarized guinea-pig aortic strips and nonvascular ileum longitudinal smooth muscle. The relaxation of nonvascular tissues, and in particular of ileum longitudinal smooth muscle, may cause undesired side effects such as constipation when treating a patient with an LTCC blocker as antihypertensive. In vascular smooth muscle, only two compounds (**30** and **34**) were active; the uniqueness of **34** is the naphthyl group in place of the phenyl at C8, whereas the uniqueness of **30** is the chlorine substitution at the fused benzothiazino ring, with a certain resemblance to the diltiazem derivative, clentiazem. 

**Table 1:** Cardiac activity of compounds **24**–**40**.

	Left Atrium			Right Atrium		
	Negative Inotropy			Negative Chronotropy		
Compd	Activity *^a^* (M ± SEM)	EC_50_ *^b^* (µM)	95% conf lim (×10^−6^)	Activity *^c^* (M ± SEM)	EC_30_ *^b^* (µM)	95% conf lim (×10^−6^)
**24**	78 ± 3.5	0.79	0.70–0.85	94 ± 5.6 *^d^*	0.07	0.064−0.075
**25** *^e^*	77 ± 1.7 *^d^*	0.04	0.03−0.05	5 ± 0.2 *^f^*		
**26** *^g^*	76 ± 2.5 *^h^*	0.022	0.015−0.031	4 ± 0.1		
**27** *^e^*	81 ± 2.9 *^i^*	0.63	0.45–0.80	20 ± 1.1 *^i^*		
**28** *^g^*	81 ± 3.9 *^d^*	0.013	0.0085–0.018	7 ± 0.2 *^f^*		
**29** *^g^*	75 ± 1.7 *^d^*	0.0060	0.0042–0.0087	7 ± 0.3 *^f^*		
**30**	87 ± 2.3	0.36	0.26–0.48	2 ± 0.2		
**31**	82 ± 2.6 *^i^*	0.44	0.32–0.61	2 ± 0.1 *^f^*		
**32**	76 ± 2.7	3.67	2.91–4.12	56 ± 3.1	5.06	4.38−5.91
**33**	88 ± 0.4 *^i^*	5.25	3.29–8.36	52 ± 1.9	6.25	5.14−7.36
**34**	95 ± 1.3 *^i^*	0.057	0.040–0.082	7 ± 0.1		
**35**	88 ± 3.6	0.13	0.090–0.17	28 ± 1.6 *^f^*		
**36**	85 ± 3.2	0.039	0.024–0.064	34 ± 1.4		
**37**	85 ± 4.2	1.11	0.76–1.61	16 ± 0.7		
**38**	68 ± 2.3	4.23	3.45–5.26	29 ± 1.6		
**39**	95 ± 3.6 *^f^*	1.08	0.67–1.93	5 ± 0.3		
**40**	92 ± 3.4 *^f^*	0.76	0.52–1.12	16 ± 0.9		

*^a^* Decrease in developed tension on isolated guinea-pig left atrium at 10^−^^5^ M, expressed as percent changes from the control (n = 5–6). The left atria were driven at 1 Hz. The 10^−^^5^ M concentration gave the maximum effect for most compounds; *^b^* Calculated from log concentration-response curves (Probit analysis by Litchfield and Wilcoxon [28] with n = 6–7). When the maximum effect was <50%, the EC_50_ ino., EC_30_ chrono., values were not calculated; *^c^* Decrease in atrial rate on guinea-pig spontaneously beating isolated right atrium at 10^−^^5^ M, expressed as percent changes from the control (n = 7–8). The 10^−^^5^ M concentration gave the maximum effect for most compounds. Pretreatment heart rate ranged from 165 to 190 beats/min; *^d^* At the 10^−^^6^ M; *^e^* From reference 12; *^f^* At the 10^−^^4^ M; *^g^* From reference 26; *^h^* At the 5 × 10^−^^6^ M; *^i^* At the 5 × 10^−^^5^ M. 95% conf lim stands for 95% confidence limit.

**Table 2:** Relaxant activity of compounds **24**–**40** on K^+^-depolarized guinea pig vascular and nonvascular smooth muscle.

Compd	Aorta			Ileum		
	Activity *^a^* (M ± SEM)	IC_50 _*^b^* (μM)	95% conf lim (×10^−6^)	Activity *^a^* (M ± SEM)	IC_50 _*^b^* (μM)	95% conf lim (×10^−6^)
**24**	88 ± 2.3	2.6	2.2–3.1	98 ± 1.5 *^c^*	0.11	0.085−0.13
**25 *^d^***	19 ± 0.9 *^e^*			3 ± 0.2		
**26 *^f^***	28 ± 1.7			73 ± 0.2	25.94	18.97−35.45
**27 *^d^***	11 ± 0.8 *^e^*			*		
**28 *^f^***	4 ± 0.3			87 ± 1.5	8.32	6.37−10.85
**29 *^f^***	21 ± 0.9			54 ± 2.1	11.79	4.33−18.21
**30**	57 ± 3.3	25.82	13.54–49.24	90 ± 1.6 *^e^*	19.73	8.25–23.12
**31**	10 ± 0.9			58 ± 1.4	13.99	10.67–18.34
**32**	43 ± 2.3			85 ± 1.6 *^g^*	3.26	2.58–4.11
**33**	31 ± 2.9			64 ± 2.4 *^g^*	5.51	4.36–6.96
**34**	70 ± 2.9	29.88	18.35–31.07	98 ± 1.0	13.22	10.67–16.38
**35**	26 ± 2.5			66 ±2.4	34.36	27.72–42.59
**36**	27 ± 1.6			86 ± 1.7	16.63	13.63–20.27
**37**	45 ± 0.7			59 ± 0.1 *^g^*	7.87	6.73–9.20
**38**	31 ± 0.7			78 ± 2.4 *^g^*	3.49	2.77–4.42
**39**	22 ± 2.1			91 ± 3.4	21.35	17.26–26.40
**40**	19 ± 0.9			52 ± 0.3 *^g^*	9.97	8.39–10.85

*^a^* Percent inhibition of calcium-induced contraction on K+-depolarized (80 mM) guinea pig aortic strips and longitudinal smooth muscle (at 10^−4^ M). The 10^−4^ M concentration gave the maximum effect for most compounds respectively; *^b^* Calculated from log concentration− response curves (Probit analysis by Litchfield and Wilcoxon [28] with n = 6–7). When the maximum effect was <50%, the IC_50_ values were not calculated; *^c^* At the 10^−^^6^ M; *^d^* From reference 12; *^e^* At the 5 × 10^−^^5^ M; *^f^* From reference 26; *^g^* At the 10^−^^5^ M; * Not tested. 95% conf lim stands for 95% confidence limit.

In nonvascular smooth muscle, all of the tested molecules (with **25** as the only exception) show activity in the ileum, but diltiazem remains more potent than all of them. With these experimental conditions any relaxant activity of nonvascular smooth muscle, if present, would be attributed to a calcium antagonist activity.

#### 2.2.2. Electrophysiology and Binding Data

It is well established that contraction of smooth muscle is initiated, and to a lesser extent maintained, by a rise in the concentration of free Ca^2+^ in the cell cytoplasm [29]. This activator Ca^2+^ can either enter from the extracellular space through a variety of Ca^2+^ permeable ion channels—the best-characterized Ca^2+^ entry pathway utilizes LTCC—or be released by the sarcoplasmic reticulum [30,31,32].

Although all of the molecules contain the same scaffold, only two of them exhibit a vasorelaxant effect (compounds **30** and **34**) and only two exhibit a chronotropic effect (compounds **32** and **33**). Thus, we selected **30** and **32** for in-depth studies. The effects of compounds **30** and **32** on vascular smooth muscle have been presently investigated under experimental conditions that allow for the identification of blockers of these LTCC. At first, we tested compounds **30** and **32** on single smooth muscle cells isolated from the rat tail main artery, in order to provide direct evidence of their LTCC blocking activity.

**Figure 2:**
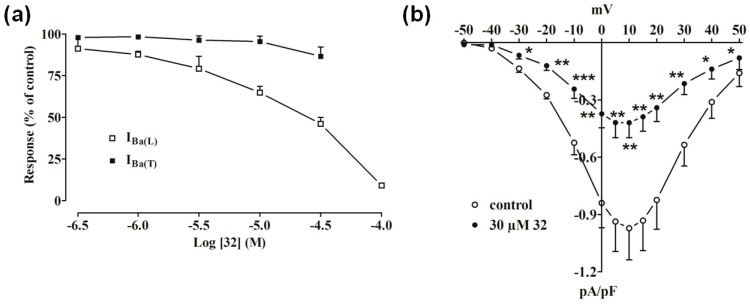
Effect of compound **32** on I_Ba_ in single tail artery myocytes. (**a**) Concentration-dependent effect of **32** at the peak of I_Ba(L)_ and I_Ba(T)_ trace. On the ordinate scale, response is reported as percentage of control. Data points are mean ± SEM (n = 3–6); (**b**) Current-voltage relationships, recorded from a V_h_ of –80 mV, constructed prior to the addition (control) and in the presence of 30 µM **32**. Data points are mean ± SEM (n = 6). * *p* < 0.05, ** *p* < 0.01, *** *p* < 0.001* vs.* control, Student’s *t* test for paired samples.

Compound **32** inhibited I_Ba(L)_ (Ba^2+^ current through L-type Ca^2+^ channels), measured at 0 mV from a holding potential (V_h_) of −80 mV, in a concentration-dependent manner (Figure 2a) with an estimated IC_50_ of 30 µM and accelerated its inactivation kinetics (data not shown). On the contrary, I_Ba_ recorded at −40 mV from V_h_ of −80 mV (an indicator of T-type currents [I_Ba(T)_ (Ba^2+^ current through T-type Ca^2+^ channels)]) was not significantly modified by **32** up to a concentration of 30 µM. The current-voltage relationship (Figure 2b) shows that **32** significantly decreased the peak inward current in the range between −30 mV and 50 mV without varying the apparent maximum at about 10 mV and the threshold at about −30 mV.

**Figure 3:**
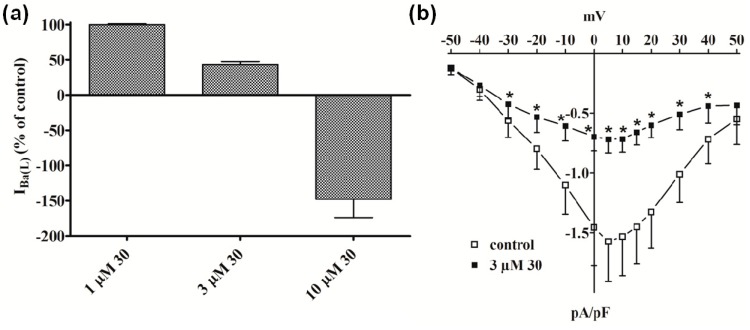
Effect of compound **30** on I_Ba(L)_ in single tail artery myocytes. (**a**) Concentration-dependent effect of **30** at the peak of I_Ba(L)_ trace. On the ordinate scale, response is reported as percentage of control. Data points are mean ± SEM (n = 4–5); (**b**) Current-voltage relationships, recorded from a V_h_ of −80 mV, constructed prior to the addition (control) and in the presence of 3 µM **30**. Data points are mean ± SEM (n = 5). * *p* < 0.05* vs.* control, Student’s *t* test for paired samples.

Similar results were obtained with **30**, which was ineffective on I_Ba(T)_ (data not shown), decreased I_Ba(L)_ by about 50% at 3 µM (Figure 3a), accelerated its decay (data not shown), and reduced the current-voltage relationship in the range between −30 mV and 40 mV (Figure 3b). Noticeably, a strong outward current (possibly ascribable to Cs^+^ flowing through LTCC) appeared when myocytes were challenged with 10 µM **30**.

High K^+^-induced contraction of aorta strips is the result of an increased Ca^2+^ influx through LTCCs and is specifically inhibited by Ca^2+^-antagonists. We found that compounds **30** and **32** antagonize high K^+^-induced contraction in a concentration-dependent manner. Therefore, this inhibition might be interpreted as a consequence of the blockade of LTCC. The electrophysiological data presented here directly confirm this hypothesis, since both compounds inhibited I_Ba(L)_ in rat tail artery myocytes; their potency, however, was one order of magnitude lower than that exhibited in inhibiting high K^+^-induced contractions. This observation suggests that the vasorelaxing activity, lower as one should expect from the LTCC-blocking effect, is likely limited either by the diffusion of the drugs into the whole tissue or by the *t*_0.5_ of the agents. Furthermore, slight differences in L-type Ca^2+^ channels expressed in the two tissues examined might explain the incomplete matching of data from the two experimental settings.

Compounds **30**- and **32**-induced inhibition of I_Ba(L)_, observed at 0.067 Hz, a frequency that allows full recovery between pulses from Ca_v_1.2 channel inactivation in rat tail artery myocytes [33], was tonic in nature and developed independently of channel activation [32]. This is interpreted as a consequence of the selective inhibition of the resting channel. In addition, the faster LTCC inactivation kinetics observed in the presence of both compounds (data not shown), reported also for other LTCC blockers such as dihydropyridines and phenylalkylamines [34], likely indicates that the drugs exert an open channel inhibition [35].

Binding assays on rat cardiomyocytes were carried out in order to establish whether they could displace [^3^H]diltiazem from its binding site. Surprisingly, **32** (1 nM–100 µM) did not affect [^3^H]diltiazem binding and this along with its low vasorelaxant activity (compared to diltiazem) was considered a sufficient reason for not further investigating the mechanisms underlying **30**-mediated chronotropic activity. Compound **30** even revealed a peculiar behavior which was comparable in part to that observed for its analogue **25**. The latter compound, in fact, displayed a complex interaction with the benzothiazepine receptor since it either stimulated or inhibited the binding of the labeled calcium antagonist at low (0.1 nM–1 μM) or high (10–100 μM) concentrations, respectively [36]. Compound **30** was able to potentiate [^3^H]diltiazem binding in a wide range of concentrations (1 nM–1 µM) while it was inactive in the range 1–100 µM.

### 2.3. 3D-QSAR Model for the LTCC Negative Inotropic Activity of the Oxadiazolothiazinones

Quantitative structure-activity relationship (QSAR) models relate a set of X-variables to the potency of the response variable, Y; 3D-QSAR methods require three-dimensional molecular structures and use as X-variables various descriptors quantifying electronic, geometric, or steric properties, all calculated over aligned 3D-structures. In the GRID/Golpe 3D-QSAR approach, energy values of the GRID molecular interaction fields (MIF) are used as descriptors [37]. It is well-established that one of the major drawbacks of 3D-QSAR modeling was to define the molecular alignment, especially for compounds with different scaffolds. Some years ago some of us developed a QSAR model for LTCC negative inotropic activity, based on several oxadiazolothiazinones and a few benzothiazepine-like compounds; we applied the method named GRIND, that does not directly use the MIF but encodes the energy information into alignment-independent descriptors [12].

From a dynamic perspective, *in silico* models, including QSAR models, may be updated as soon as new biological data and/or new *in silico* techniques become available. Recently, we decided to build a new 3D-QSAR model—to be used in the series optimization—solely based on oxadiazolothiazinones and the GRID/3D-QSAR approach. We have enlarged the diversity of the oxadiazolothiazinones and achieved novel potent compounds; in addition, the GRID/3D-QSAR modeling has been revitalized by software that automatically aligns several molecules, even those which are structurally diverse [38].

We assembled a training set of 29 oxadiazolothiazinones, all tested with the exact same experimental conditions. About half of them have the hydroxyl group at C8, while the others an OR group, with R being either a short alkyl chain or CH_2_C_6_H_11_. Eight molecules of the set are from [11], seven molecules from [12], three molecules from [26], and 11 molecules are new (Figure 1). Data and structures are reported as Supplementary Materials.

**Figure 4:**
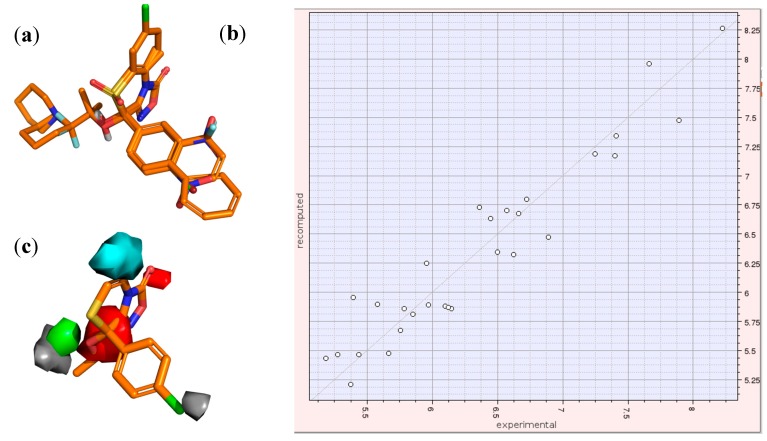
The three steps of 3D-QSAR model: (**a**) alignment of the structures; (**b**) graphical analysis of the IVPLS model; (**c**) inspection of the MIF/pseudofields together with the 3D-structure of the active molecules in order to understand the key regions for the activity. Pseudofields colours of section (c): hydrophobic = green; halogen = grey; aromatic = cyan; H-bond acceptor = red.

These 29 molecules were subjected to 3D-QSAR modeling with the software FLAP [39,40], as described in the Experimental section. The aligned structures are reported in Figure 4a. Once superimposed, values of the GRID MIF compose the X-matrix of the QSAR model: the interaction with the GRID probe H describes the molecular shape, while the probes DRY and N1 mimic the hydrophobic and H-bonding interactions (in which the ligand atoms act as H-bonding acceptors and protein atoms as donors), respectively. 

The X-matrix includes also pseudofields, that are a sort of MIF-projection onto the molecular structure. For the dependent variable for the negative inotropic activity, we used the pEC_50_ of the potency, expressed as molar concentration. To align molecules, we used a method based on molecular graphs. Finally, as regression method we used the IVPLS, that stands for Iterative Variable Simplification Partial Least Squares. This multivariate statistical method is able to improve the predictive ability of the regression models with a dimension-wide variable simplification [41].

We obtained a model composed of one latent variable, characterized by excellent correlation coefficients for objects recalculation (R^2^ = 0.91). Since all the compounds with very high or very low potency values were part of the training set, we considered misleading an external validation based only on compounds with mid-value potency. So, we carried out an internal validation, with the leave-one-out method, that gave a good objects prediction (Q^2^ = 0.56).

The plot recomputed *vs* experimental is reported in Figure 4b. In the mentioned IVPLS model, the variables with the highest impact on the activity are those corresponding to the largest coefficients. The coefficients of these variables can be plotted as isocontour surfaces as in Figure 4c, where we report the pseudofields of the 3D-QSAR model. Key regions are: an aromatic region (cyan), corresponding to the benzofused ring at C5–C6; a hydrophobic region (green) corresponding to the OR alkyl chain; two halogen regions (grey) corresponding to the *para*-position of the phenyl at the C8 and to the alkyl chain; and two H-bonding accepting regions (red), one corresponding to the nitrogen and OH/OR group, and the other to the two oxygen atoms of the oxadiazol-3-one moiety.

**Figure 5:**
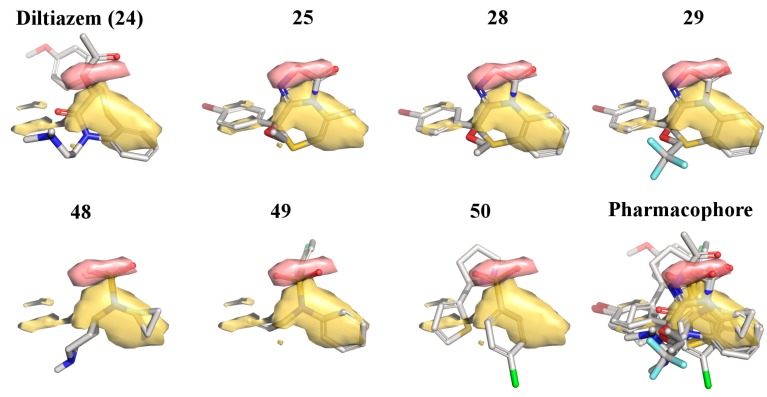
Pharmacophore model obtained with seven molecules: diltiazem (**24**), three oxadiazolothiazinones (**25**, **28**, and **29**), and three compounds from [36] (**48**, **49**, and **50**). Pseudofield colours: hydrophobic = yellow; H-bond acceptor = red.

In the past, we used the *R*-form of **25** as a template for ligand-based virtual screening [36], that allowed us to identify new chemotypes active as LTCC blockers. Here, we report how the most potent compounds that resulted from that screening were used together with diltiazem and the most potent oxadiazolothiazinones to build a pharmacophore model valid for small-molecules with LTCC negative inotropic activity (Figure 5). 

**Figure 6:**
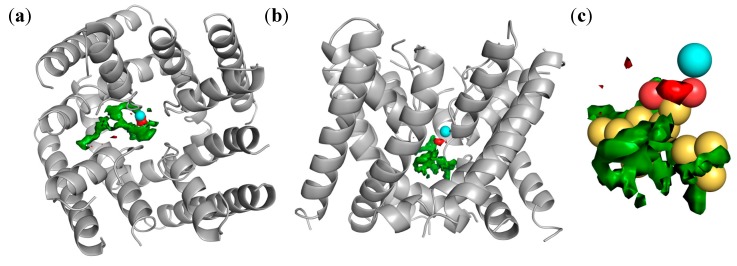
(**a**,**b**): Two views of the 3D-structure of LTCC (homology model by Tikonov and Zhorov [43]), in which one calcium ion is shown as a cyan sphere, whereas green and red surfaces represent GRID molecular interaction fields obtained within the pocket with the hydrophobic probe (DRY, field shown at −2.0 kcal/mol) and the carbonyl oxygen probe (O, field shown at −5.0 kcal/mol), respectively; (**c**) The pharmacophore, represented by yellow spheres (hydrophobic atoms) and red spheres (H-bonding accepting atoms), is reported docked within the LTCC cavity.

With the FLAPpharm module of FLAP [42] we aligned all of the mentioned structures: the alignment of the pharmacophoric points is maximized through a scoring function that is a weighted sum of the shape, hydrophobic and hydrogen-bond MIF similarities. For the aligned molecules we can visualize the pseudofields (Figure 5: hydrophobic = yellow; H-bond acceptor = red), but also the common location of the pharmacophoric points, encoded into a pseudomolecule that can be used as a ligand in a docking procedure (Figure 6), to rationalize structure-activity relationships and study the interaction with the protein (as described below), or as a template in a ligand-based virtual screening (that is beyond the scope of the present paper).

We used a homology model [43] of the LTCC protein as a target for structure-based docking of this pseudomolecule. Figure 6a,b show two different views of the pseudomolecule once docked into the LTCC pocket; in Figure 6c the pharmacophore is shown with the sphere-representation in order to highlight the interaction with the calcium ion: yellow spheres represent hydrophobic atoms, whereas the two red spheres the H-bonding accepting atoms. In the interaction with the protein (shown in grey), hydrophobic features interact in correspondence to the green MIF of the protein, whereas a pair of H-bonding accepting atoms, a few Angstroms apart from each other, are likely able to act as chelating agents for the proximal calcium ion (cyan sphere).

Our findings are in agreement with the results from Tikonov and Zhorov [43], who published the homology model for LTCC that we used here, and reported about a ternary complex between diltiazem, the calcium ion, and the protein. The comparison of the docked pharmacophore with diltiazem as within the LTCC protein as proposed by Tikonov and Zhorov is reported as Supplementary Material. The results of the pharmacophore-docking let us hypothesize the formation of a similar ternary complex between each ligand, the calcium ion, and the protein.

In both the 3D-QSAR model (Figure 4) and the pharmacophore-docking (Figure 5 and Figure 6) the oxadiazolone moiety played a major role, with the oxygen atoms key for the interaction with the positively charged calcium ion. In the next section we provide further evidence of the relevance of this scaffold in biological interactions: the docking analysis of a set of oxadiazolothiazinones into P-glycoprotein 1.

### 2.4. Docking of Oxadiazolothiazinones into Human P-glycoprotein 1 Homology Model

To study the binding modes of some oxadiazolothiazinones **25**–**27** to human Pgp-170, we adopted a model of the Nucleotide binding domain 1 (MONBD1), as described in [14]. We obtained a good agreement between *in silico* predictions (docked solutions of compounds) and* in vitro* results (MDR1-resistant ovarian A2780/DX3 cancer cells).

The reversal of multidrug resistance was studied in multidrug resistant A2780/DX3 cells. The MTT assay was used to define the activity of doxorubicin with or without the modulators. In our experimental conditions, **27** caused a 76% increase accumulation of doxorubicin, compared to doxorubicin alone, with values of 0.60 ± 0.19 μM *vs.* 2.10 ± 0.42 μM. The other two compounds, **25** and **26**, did not cause a pharmacologically significant increase of doxorubicin activity (IC_50_ reduction: **25**: −20%; **26**: −14%). In agreement with experimental* in vitro* data, we observed significant differences in the docking results, with favorable clusterization and a good score for **27** and unfavorable clusterization observed for **25** and **26**.

**Figure 7:**
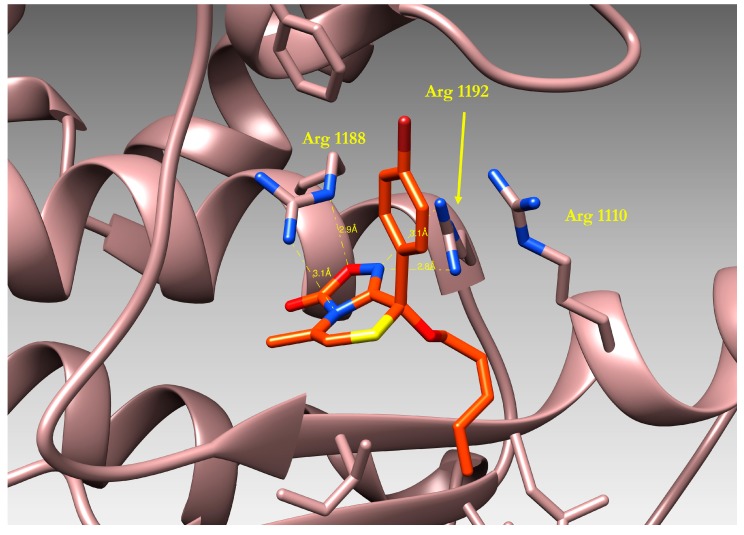
3D-structure of compound **27** bound to the Nucleotide Binding Domain1 (NBD1) of human ABCB1, as resulting from docking simulations.

The binding mode of compound **27** is reported in Figure 7: a visual inspection of the complex formed by the ligand and the protein model evidenced the importance of the oxadiazolothiazinone moiety which is involved in different interactions with the protein binding site. Hydrogen bonds are formed with the guanidyl groups of Arginines 1110, 1188 and 1192, and with the OH atom of Ala 1185. On the whole, our data* in vitro* data confirmed [13] that some of the oxadiazolothiazinones supposed to inhibit the human Pgp-170 were able to potentiate doxorubicin activity in A2780/DX3 resistant cells and, furthermore, were active at concentrations unable to inhibit cell proliferation. Moreover, they were not able to down-regulate the membrane MDR1 thus further suggesting a mechanism of action directly involving the inhibition of the MDR1 transporter.

**Figure 8:**
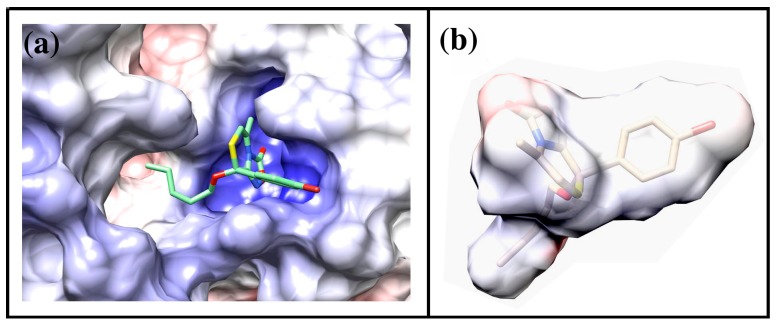
(**a**) Representation of Molecular Electrostatic Potential for the P-glycoprotein and (**b**) compound **27**. In both cases the surface is colored according to its electrostatic potential (blue = positively charged, red = negatively charged). Figure is drawn using the program Chimera [44], electrostatic potential is calculated using the AMBER force field [45].

We calculated the Molecular Electrostatic Potential (MEP) for the protein and for one of the oxadiazolothiazinones (compound **27**), represented in Figure 8. The presence of arginines in the binding pocket of P-glycoprotein determines a positively charged environment (blue region in Figure 8a) where the oxadiazolone moiety of **27** (red fields in Figure 8b) is likely to interact.

## 3. Experimental

### 3.1. General Information 

Compounds **38** [26], **41b** [46], **44** [19], and **45** [18], were synthesized according to the respective literature procedures. Compounds **41a**, **c**, and **d** were commercially available. Melting points were determined using a Büchi apparatus B 540 and are uncorrected. ^1^H- and ^13^C-NMR spectra were recorded on a Varian Gemini 300 Instrument in the Fourier transform mode at 25 (±0.5 °C) in DMSO-*d*_6_. Chemical shifts (δ) are in parts per million (ppm) from tetramethylsilane and coupling constants are in Hertz. ^1^H-NMR and ^13^C-NMR spectra are reported as Supplementary Material. ESI mass spectra were obtained on a micromass ZMD Waters instrument (30 V, 3.2 kV, isotope observed ^79^Br). EI mass spectra were recorded on a VG70 70E apparatus. Solvents of reaction were removed under reduced pressure. Silica gel plates (Merck F_254_) and silica gel 60 (Merck 230–400 mesh) were used for analytical TLC and for column chromatography, respectively. All new compounds gave satisfactory microanalyses (C, H, halogen, and N).

### 3.2. Synthesis

*2-(4-Bromophenyl)-7-chloroimidazo[2,1-b][1,3]benzothiazole* (**42a**). 2,4'-Dibromoacetophenone (5.0 g, 18 mmol) was added to a solution of 2-amino-6-chlorobenzothiazole (**41a**, 18 mmol) in ethanol (30 mL) and triethylamine (18 mmol). After refluxing (30 min), **42a** was separated by filtration (yield 30%; mp 244.0–244.8 °C from ethanol). ^1^H-NMR: δ 7.62–7.66 (3H, m, H-5, and HAr); 7.56 (1H, dd, *J* = 8.8 and *J* = 1.8 Hz, H-7); 7.80 (2H, part XX' of the system AA'XX', HAr); 7.98 (1H, d, *J* = 8.8 Hz, H-8); 8.23 (1H, d, *J* = 1.8 Hz, H-5); 8.84 (1H, s, H-3). ^13^C-NMR: δ 109.8; 114.5; 120.1; 124.6; 126.6; 126.8; 129.1; 130.7; 131.0; 131.7; 132.9; 145.3; 147.7. EI: *m/z* (%) 366-364-362 (M^+^, 100). 389-387-385 (M+23). HMRS (^79^Br, ^35^Cl isotopes): *m/z* calcd for C_15_H_8_BrClN_2_S: 361.9280; found: 361.9283.

*6-(4-Bromophenyl)imidazo[2,1-b][1,3]thiazole* (**42b**). 2,4'-Dibromoacetophenone (5.0 g, 18 mmol) was added to a solution of 2-aminothiazole (**41b**, 18 mmol) in CHCl_3_ (30 mL) and the mixture was refluxed for 2 h. After cooling, the separated crystals were collected by filtration, added of ethanol (20 mL) and refluxed with 2 M HCl (20 mL) for 2 h. The mixture was treated with saturated NH_4_OH solution until basic and the collected precipitate was crystallized from ethanol to give **42b** (yield 81%; mp 180.1–180.8 °C; lit. [47] 182 °C, lit. [48] 179–180 °C). ^1^H-NMR: δ 7.28 (1H, d, *J* = 5.3 Hz, H-3); 7.57 (2H, part AA' of the system AA'XX', HAr); 7.79 (2H, part XX' of the system AA'XX', HAr); 7.94 (1H, d, *J* = 5.3 Hz, H-2); 8.27 (1H, s, H-5). ^13^C-NMR: δ 109.8; 113.4; 119.8; 120.0; 126.7; 131.5; 133.5; 145.1; 149.4. HMRS (^79^Br isotope): *m/z* calcd for C_11_H_7_BrN_2_S: 277.9513; found: 277.9514.

*6-(4-Bromophenyl)-3-ethylimidazo[2,1-b][1,3]thiazole* (**42c**). 2,4'-Dibromoacetophenone (5.0 g, 18 mmol) was added to a solution of 2-amino-4-ethylthiazole (**41c**, 18 mmol) in acetone (30 mL) and the mixture was refluxed for 2 h. After cooling, the deposited crystals were collected by filtration, added of ethanol (20 mL) and refluxed with 2 M HCl (20 mL) for 2 h. The mixture was treated with saturated NH_4_OH solution until basic and the collected precipitate was crystallized from ethanol to give **42c** (yield 42%; mp 145.2–146.1 °C). ^1^H-NMR: δ 1.30 (3H, t, *J* = 7.5 Hz, CH_3_); 2.80 (2H, dq, *J* = 7.5 Hz, *J* = 1.3 Hz, CH_2_); 6.94 (1H, t, *J* = 1.3 Hz, H-2); 7.60 (2H, part AA' of the system AA'XX', HAr); 7.81 (2H, part XX' of the system AA'XX', HAr); 8.43 (1H, s, H-5). ^13^C-NMR: δ 10.7; 20.4; 107.6; 108.7; 120.1; 126.7; 131.6; 133.6; 133.9;143.8; 148.6. ESI: *m/z* 306 (M); 329 (M + 23). HMRS (^79^Br isotope): *m/z* calcd for C_13_H_11_BrN_2_S: 305.9826; found: 355.9828. 

*3-Methyl-6-(2-naphthyl)imidazo[2,1-b][1,3]thiazole* (**42d**). 2-Bromoacetonaphthone (5.23 g, 21.0 mmol) was added to a solution of 2-amino-4-methylthiazole (**41d**, 2.0 g, 18.0 mmol) in acetone (40 mL) and the mixture was refluxed for 2 h, then the solvent was distilled off. The crude was added of ethanol (30 mL) and refluxed with 2 M HCl (30 mL) for 3 h. The mixture was treated with saturated NH_4_OH solution until basic, the precipitate was collected by filtration, whereas the solution was extracted with CHCl_3_ (4 × 50 mL) and the organic layer was dried on Na_2_SO_4_. Removal of the solvent left a solid which was added to the first portion and purified by flash chromatography (ethyl acetate:petroleum ether = 1:5 → 1:0 v/v as eluant) to give the desired compound, which was purified by crystallization from ethanol (yield 66%; mp 160.8–161.4 °C). ^1^H-NMR (500 MHz, DMSO): δ 8.40 (2H, s, H-3 and H-Ar); 8.01 (1H, pd, H-Ar); 7.93 (2H, pd, H-Ar); 7.89 (1H, pd, H-Ar); 7.51 (1H, pt, H-Ar); 7.47 (1H, pt, H-Ar); 6.92 (1H, s, H-6); 2.45 (3H, s, CH_3_). ^13^C-NMR (125 MHz, DMSO): δ 148.8; 146.2; 133.3; 132.2; 131.8; 128.2; 128.1; 127.8; 127.6; 126.4; 123.5; 122.6; 108.6; 107.1; 12.8. EI: *m/z* (%) 264 (100); 153 (10); 69 (14). ESI: *m/z* 287 (M + 23). HMRS:* m/z* calcd for C_16_H_12_N_2_S: 264.07212; found: 264.0722.

General Procedure for the synthesis of 5-nitrosoimidazo[2,1-b][1,3]thiazole derivatives **43a**–**d**. 

A solution of sodium nitrite (8.7 mmol) in water (10 mL) was added, under cooling and stirring, to a solution of the appropriate imidazo[2,1-*b*][1,3]thiazole **42** (4 mmol) in acetic acid (20 mL). After 6–30 h (TLC analysis) and a new addition of sodium nitrite (8.7 mmol) at room temperature (or at 50 °C in the instance of **43a**), the mixture was neutralized with 2 M NaOH and the green precipitate was collected and crystallized from ethanol.

*2-(4-Bromophenyl)-7-chloro-3-nitrosoimidazo[2,1-b][1,3]benzothiazole* (**43a**). Yield: 90%; mp 256.4–257.1 °C. ^1^H-NMR: δ 7.73 (1H, dd, *J* = 9.1 Hz, *J* = 2.1 Hz, H-6); 7.85 (2H, part AA' of the system AA'XX', HAr); 8.36 (1H, ps, H-8); 8.43 (2H, part XX' of the system AA'XX', HAr); 8.76 (1H, d, *J* = 9.1 Hz, H-5). HMRS (^79^Br, ^35^Cl isotopes): *m/z* calcd for C_15_H_7_BrClN_3_OS: 390.9182; found: 390.9184.

*6-(4-Bromophenyl)-5-nitrosoimidazo[2,1-b][1,3]thiazole* (**43b**). Yield: 90%; mp 218.0–219.0 °C (lit. [49] 220 °C). ^1^H-NMR: δ 7.67 (1H, d, *J* = 4.3 Hz, H-3); 7.83 (2H, part AA' of the system AA'XX', HAr); 8.46 (1H, d, *J* = 5.3 Hz, H-2); 8.4 (2H, part XX' of the system AA'XX', HAr). ^13^C-NMR: δ 120.6; 121.4; 125.6; 130.7; 131.3; 132.2; 156.2; 156.39; 159.3. HMRS (^79^Br isotope): *m/z* calcd for C_11_H_6_BrN_3_OS: 306.9415; found: 306.9415.

*6-(4-Bromophenyl)-3-ethyl-5-nitrosoimidazo[2,1-b][1,3]thiazole* (**43c**). Yield: 76%; mp 188.0–190.0 °C. ^1^H-NMR: δ 1.06 (3H, t, *J* = 7.3 Hz, CH_3_); 3.03 (2H, q, *J* = 7.3 Hz, *J* = 1.3 Hz, CH_2_); 7.19 (1H, s, H-2); 7.79 (2H, part AA' of the system AA'XX', HAr); 8.32 (2H, part XX' of the system AA'XX', HAr). ^13^C-NMR: δ 13.4; 23.7; 112.9; 125.23; 130.9; 131.9; 132.0; 138.8; 156.1;159.9; 161.3. ESI: *m/z* 334 (M); 357 (M + 23). HMRS (^79^Br isotope): *m/z* calcd for C_13_H_10_BrN_3_OS: 334.9728; found: 334.9729.

*3-Methyl-6-(2-naphthyl)-5-nitrosoimidazo[2,1-b][1,3]thiazole* (**43d**). Yield: 90%; mp 216.4–217.2 °C. ^1^H-NMR (500 MHz, DMSO): δ 9.01 (1H, s, H-Ar); 8.50 (1H, pd, H-Ar); 8.11 (1H, pd, H-Ar); 8.08 (1H, pd, H-Ar); 8.01 (1H, pd, H-Ar); 7.65 (1H, pt, H-Ar); 7.61 (1H, pt, H-Ar); 7.18 (1H, s, H-6); 2.60 (3H, s, CH_3_). ^13^C-NMR (125 MHz, DMSO): δ 161.4; 160.5; 156.5; 133.9; 133.1; 132.6; 130.8; 129.2; 129.1; 128.2; 127.8; 126.8; 126.4; 113.7; 17.0. EI: *m/z* (%) 293 (1000); 292 (30); 278 (21); 277 (91); 276 (84); 266 (22); 264 (53); 264 (33); 262 (26); 244 (29); 239 (20); 238 (37); 179 (26); 164 (34); 153 (47); 139 (25); 127 (33); 72 (30); 71 (39). ESI: *m/z* 316 (M + 23). HMRS:* m/z* calcd for C_16_H_11_N_3_OS: 293.06228; found: 293.0623.

General procedure for the synthesis of hydroxy[1,2,4]oxadiazolo[3,4-c][1,4]thiazinones **30**, **34**, **35**, and **37**. A suspension of the appropriate nitroso derivative **43** (3 mmol) in ethanol (25 mL) was refluxed under stirring with 2 M HCl (2.5 mL) for 2–4 h. Removal of the solvent left a solid which was purified by flash-chromatography to give the desired compound.

*4-(4-Bromophenyl)-7-chloro-4-hydroxy-4H-[1,2,4]oxadiazolo[3,4-c][1,4]benzothiazin-1-one* (**30**). Eluant mixture: ethyl acetate-petroleum ether = 1:5 v/v. Yield: 53%; mp 201.1–201.9 °C from toluene. ^1^H-NMR: δ 7.59–7.66 (3H, m, H-6 or H-8 and part AA' of the system AA'XX', HAr); 7.70–7.75 (3H, m, H-8 or H-6 and part XX' of the system AA'XX', HAr); 8.21 (1H, d, *J* = 8.8 Hz, H-9); 8.70 (1H, s exch., OH). ^13^C-NMR: δ 77.8; 119.4; 123.1; 125.3; 127.7; 128.3; 128.6; 129.1; 131.1; 131.3; 135.3; 155.0; 155.3. EI: *m/z* (%) 368–366 (7), 229–227 (13); 185–183 (100), 157–155 (30). HMRS (^79^Br isotope): *m/z* calcd for C_15_H_8_BrClN_2_O_3_: 409.9127; found: 409.9128.

*8-Hydroxy-5-methyl-8-(2-naphthyl)-8H-[1,2,4]oxadiazolo[3,4-c][1,4]thiazin-3-one* (**34**). Eluant mixture: ethyl acetate-petroleum ether = 1:4 v/v. Yield: 45%; mp 155.6–156.7 °C from ethanol. ^1^H-NMR (400 MHz, DMSO): δ 8.36 (1H, s, OH); 8.25 (1H, s, H-Ar); 8.04–7.94 (3H, m, H-Ar); 7.70 (1H, pd, H-Ar); 7.62–7.55 (2H, m, H-Ar); 6.26 (1H, s, H-6); 2.44 (3H, s, CH_3_). ^13^C-NMR (100 MHz, DMSO): δ 155.8; 155.0; 134.3; 132.9; 132.1; 128.6; 128.4; 127.8; 127.5; 127.1; 126.7; 126.3; 124.7; 103.5; 77.0; 16.7. EI: m/z (%) 312 (5); 173 (11); 172 (92); 156 (13); 155 (99); 128 (16); 127 (199); 126 (19); 81 (21); 77 (12); 69 (42). ESI: *m/z* 335 (M + 23). HMRS:* m/z* calcd for C_16_H_12_N_2_O_3_S: 312.05687; found: 312.0565.

*8-(4-Bromophenyl)-8-hydroxy-8H-[1,2,4]oxadiazolo[3,4-c][1,4]thiazin-3-one* (**35**). Eluant mixture: ethyl acetate-*n*-hexane = 1:2 v/v. Yield: 45%; mp 164.4–164.5 °C from toluene. ^1^H-NMR: Χ 6.60 (1H, d, *J* = 7.5 Hz, H-6); 7.26 (1H, d, *J* = 7.5 Hz, H-5); 7.62 (2H, part AA' of the system AA'XX', HAr); 7.68 (2H, part XX' of the system AA'XX', HAr); 8.44 (1H, s exch., OH). ^13^C-NMR: δ 77.0; 109.8; 116.2; 122.8; 129.3; 131.2; 136.4; 154.1; 154.7. EI: *m/z* (%) 326–328 (M^+^, 3), 226–224 (10), 185–183 (100), 157–155 (45); 147 (15); 91(25); 81 (19). ESI: *m/z* 349 (M + 23). HMRS (^79^Br isotope): *m/z* calcd for C_11_H_7_BrN_2_O_3_S: 325.9362; found: 325.9361.

*8-(4-Bromophenyl)-5-ethyl-8-hydroxy-8H-[1,2,4]oxadiazolo[3,4-c][1,4]thiazin-3-one* (**37**). Eluant mixture: ethyl acetate-petroleum ether = 1:3 v/v. Yield: 50%; mp 153.5–155.2 °C from EtOH/H_2_O. ^1^H-NMR: δ 1.13 (3H, t, *J* = 7.3 Hz, CH_3_); 2.87 (2H, qd, *J* = 7.3 Hz, *J* = 1.0 Hz, CH_2_); 6.23 (1H, t, *J* = 1.0 Hz, H-6); 7.58 (2H, part AA' of the system AA'XX', HAr); 7.67 (2H, part XX' of the system AA'XX', HAr); 8.25 (1H, s exch., OH). ^13^C-NMR: δ 12.0; 23.0; 76.2; 104.3; 122.7; 129.2; 131.1; 134.2; 136.3; 154.6; 155.8. ESI: *m/z* 354 (M – 1). HMRS (^79^Br isotope): *m/z* calcd for C_13_H_11_BrN_2_O_3_S: 353.9674; found: 353.9677.

General procedure for the synthesis of alkoxy-[1,2,4]oxadiazolo[3,4-c][1,4]thiazinones **31**, **36**, **46**, and **47**. A suspension of the appropriate hemithioacetal (**38**, **35**, **44**, or **45**, respectively; 1.3 mmol) in dry toluene (40 mL) was refluxed for 4 h under stirring with 13.0 mmol of the opportune alcohol and in the presence of a catalytic amount of *p*-toluenesulfonic acid (ca. 0.5 mmol). The reaction mixture was cooled at room temperature and washed with a saturated aqueous solution of NaHCO_3_. The organic layer was separated and the aqueous layer was extracted with toluene (3 × 40 mL). The collected organic phases were dried on Na_2_SO_4_. Removal of the solvent left a solid which was purified by flash-chromatography to give the desired compound.

*4-(4-Bromophenyl)-4-isopropoxy-4H-[1,2,4]oxadiazolo[3,4-c][1,4]benzothiazin-1-one* (**31**). Eluant mixture: ethyl acetate-petroleum ether = 1:4 v/v. Yield: 14%; mp 178.1–178.7 °C from ethanol. ^1^H-NMR: δ 0.87 (3H, d, *J* = 6.1 Hz, CH_3_); 0.90 (3H, d, *J* = 6.1 Hz, CH_3_); 3.93 (1H, sept, *J* = 6.1 Hz, CH); 7.43 (1H, t, *J* = 7.7 Hz, H-7 or H-8); 7.56 (1H, t, *J* = 7.5 Hz, H-8 or H-7); 7.66 (3H, part AA' of the system AA'XX', HAr and H-6 or H-9); 7.70 (2H, part XX' of the system AA'XX', HAr); 8.16 (1H, d, *J* = 8.0 Hz, H-9 or H-6). ^13^C-NMR: δ 23.3; 23.5; 70.2; 83.4; 118.3; 121.7; 124.0; 127.9; 128.6; 129.6; 129.9; 130.2; 131.7; 133.0; 154.5; 155.1. EI: *m/z* (%) 418–420 (M^+^, 8), 235 (28), 193 (100), 185–183 (48), 157–155 (17). ESI: *m/z* 441 (M + 23). HMRS (^79^Br isotope): *m/z* calcd for C_18_H_15_BrN_2_O_3_S: 417.9987; found: 419.9989.

*8-(4-Bromophenyl)-8-ethoxy-8H-[1,2,4]oxadiazolo[3,4-c][1,4]thiazin-3-one* (**36**). Eluant mixture: ethyl acetate-*n*-hexane = 1:5 v/v. Yield: 67%; mp 127.1–127.7 °C from toluene. ^1^H-NMR: δ 1.17 (3H, t, *J* = 6.9 Hz, CH_3_); 3.42–3.48 (1H, m, CH_2_); 3.52–3.58 (1H, m, CH_2_); 6.60 (1H, d, *J* = 7.3 Hz, H-6); 7.25 (1H, d, *J* = 7.3 Hz, H-5); 7.58 (2H, part AA' of the system AA'XX', HAr); 7.71 (2H, part XX' of the system AA'XX', HAr). ^13^C-NMR: δ 14.6; 61.1; 82.6; 108.9; 116.7; 123.3; 129.5; 131.7; 133.7; 152.2; 154.5. EI: *m/z* (%) 356–354 (M^+^, 79), 312–310 (15), 311–309 (100), 267–265 (11); 187 (12), 185–183 (95), 157–155 (70); 143 (28); 76 (28); 75 (27). ESI: *m/z* 377 (M + 23). HMRS (^79^Br isotope): *m/z* calcd for C_13_H_11_BrN_2_O_3_S: 353.9671; found: 353.9674.

*8-(2-Bromoethoxy)-8-(4-bromophenyl)-5-methyl-8H-[1,2,4]oxadiazolo[3,4-c][1,4]thiazin-3-one* (**46**). Eluant mixture: ethyl acetate-petroleum ether = 1:2 v/v. Yield: 55%; mp 141.8–142.7 °C from ethanol. ^1^H-NMR: δ 2.40 (3H, s, CH_3_); 3.64–3.68 (3H, m, CH_2_Br and CHO); 3.88–3.92 (1H, m, CHO); 6.22 (1H, s, H-6); 7.60 (2H, part AA' of the system AA'XX', HAr); 7.72 (2H, part XX' of the system AA'XX', HAr). ^13^C-NMR: δ 16.6; 31.3; 64.9; 82.0; 103.2; 123.6; 129.7; 129.8; 131.8; 132.3; 153.8; 154.7.

*8-(2-Bromoethoxy)-8-(4-chlorophenyl)-5-methyl-8H-[1,2,4]oxadiazolo[3,4-c][1,4]thiazin-3-one* (**47**). Eluant mixture: ethyl acetate-petroleum ether = 1:2 v/v. Yield: 58%; mp 111.2–112.0 °C from ethanol. ^1^H-NMR: δ 2.40 (3H, s, CH_3_); 3.64–3.70 (3H, m, CH_2_Br and OCH); 3.88–3.93 (1H, m, OCH); 6.23 (1H, s, H-6); 7.59 (2H, part AA' of the system AA'XX', HAr); 7.68 (2H, part XX' of the system AA'XX', HAr). ^13^C-NMR: δ 16.5; 31.3; 64.9; 81.9; 103.2; 128.9; 129.5; 129.8; 131.9; 134.8; 153.8; 154.6.

General procedure for the synthesis of 8-(4-halophenyl)-5-methyl-8-(2-piperidin-1-ylethoxy)-8H-[1,2,4]oxadiazolo[3,4-c][1,4]thiazin-3-ones **32** and **33**. A solution of the appropriate acetal (**46** or **47**, respectively; 1.0 mmol) in piperidine (1.5 mL) and triethylamine (0.4 mL) was refluxed for 1 h under stirring. The reaction mixture was cooled at room temperature and treated with water (10 mL), then ethyl acetate (10 mL) was added. The organic layer was separated and the aqueous layer was extracted with ethyl acetate (3 × 15 mL). The collected organic phases were dried on Na_2_SO_4_. Removal of the solvent left a solid which was purified by flash-chromatography to give the desired compound.

*8-(4-Bromophenyl)-5-methyl-8-(2-piperidin-1-ylethoxy)-8H-[1,2,4]oxadiazolo[3,4-c][1,4]thiazin-3-one* (**32**). Eluant mixture: ethyl acetate-petroleum ether = 1:1 v/v. Yield: 30%; mp 128.6–129.8 °C from *n*-hexane. ^1^H-NMR: δ 1.32–1.40 (2H, m, CH_2_); 1.43–1.52 (4H, m, 2xCH_2_); 2.20–2.38 (4H, m, 2xNCH_2_); 2.40 (3H, s, CH_3_); 2.42–2.47 (2H, m, NCH_2_); 3.37–3.42 (1H, m, OCH); 3.55–3.60 (1H, m, OCH); 6.23 (1H, s, H-6); 7.62 (2H, part AA' of the system AA'XX', HAr); 7.70 (2H, part XX' of the system AA'XX', HAr). ^13^C-NMR: δ 16.4; 23.9; 25.5; 54.3; 57.3; 62.9; 82.0; 103.4; 123.3; 129.7; 129.8; 131.6; 132.6; 154.1; 154.6. EI: *m/z* (%) 253–255 (55), 207 (17), 204 ( 22); 105 (100). ESI: *m/z* 476 (M + 23).

*8-(4-Chlorophenyl)-5-methyl-8-(2-piperidin-1-ylethoxy)-8H-[1,2,4]oxadiazolo[3,4-c][1,4]thiazin-3-one* (**33**). Eluant mixture: ethyl acetate-petroleum ether = 1:1 v/v. Yield: 28%; mp 112.5–113.0 °C from *n*-hexane. ^1^H-NMR: δ 1.31–1.39 (2H, m, CH_2_); 1.42–1.51 (4H, m, 2xCH_2_); 2.20–2.38 (4H, m, 2xNCH_2_); 2.41 (3H, s, CH_3_); 2.43–2.47 (2H, m, NCH_2_); 3.38–3.43 (1H, m, OCH); 3.55–3.61 (1H, m, OCH); 6.23 (1H, s, H-6); 7.57 (2H, part AA' of the system AA'XX', HAr); 7.69 (2H, part XX' of the system AA'XX', HAr). ^13^C-NMR: δ 16.5; 23.9; 25.6; 54.3; 57.3; 62.9; 81.9; 103.4; 128.7; 129.5; 129.7; 132.2; 134.6; 154.2; 154.6. ESI: *m/z* 430 (M + 23).

### 3.3. Functional Assays

The pharmacological profile of all compounds was derived on guinea-pig isolated left and right atria to evaluate their inotropic and/or chronotropic effects, respectively, and on K^+^-depolarized (80 mM) guinea-pig vascular (aortic strips) and nonvascular [ileum longitudinal smooth muscle (GPILSM)] to assess the calcium antagonist activity. Compounds were checked at increasing doses to evaluate: (i) the percent decrease of developed tension on isolated left atrium driven at 1 Hz (negative inotropic activity); (ii) the percent decrease in atrial rate on spontaneously beating right atrium (negative chronotropic activity); and (iii) the percent inhibition of calcium-induced contraction on K^+^-depolarized aortic strips and GPILSM (vascular and non-vascular relaxant activity, respectively). Details were already described in [12]. Data were analyzed using Student’s *t*-test and are presented as mean ± SEM [28]. Since the analyzed compounds were added in cumulative manner, the difference between the control and the experimental values at each concentration were tested for a *p* value < 0.05. The potency of drugs defined as EC_50_, EC_30_ and IC_50_ was evaluated from log concentration-response curves (Probit analysis using Litchfield and Wilcoxon [28] or GraphPad Prism^®^ software [50,51]) in the appropriate pharmacological preparations.

### 3.4. Electrophysiology Experiments

#### 3.4.1. Tail Main Artery Dissection

This investigation conforms to the Guide for the Care and Use of Laboratory Animals published by the U.S. National Institutes of Health (NIH Publication No. 85–23, revised 1996), and the animal protocols used were reviewed and approved by the Animal Care and Ethics Committee of the Università di Siena, Italy (08-02-2012). Male Wistar rats (300–400 g, Charles River Italia, Calco, Italy) were anesthetized with a mixture of Ketavet^®^ (30 mg·kg^−1^ ketamine; Intervet, Aprilia, Italy) and Xilor^®^ (8 mg·kg^−1^ xylazine; Bio 98, San Lazzaro, Italy), decapitated and exsanguinated. The tail was cut immediately, cleaned of skin and placed in physiological solution (namely external solution, containing in mM: 130 NaCl, 5.6 KCl, 10 HEPES, 20 glucose, 1.2 MgCl_2_·6 H_2_O, and 5 Na-pyruvate; pH 7.4). The tail main artery was dissected free of its connective tissue.

#### 3.4.2. Cell Isolation Procedure for I_Ba_ Recordings

Smooth muscle cells were freshly isolated from the tail main artery under the following conditions: a 5-mm long piece of artery was incubated at 37 °C for 40–45 min in 2 mL of 0.1 mM Ca^2+^ external solution containing 20 mM taurine (prepared by replacing NaCl with equimolar taurine), 1.35 mg·mL^−1^ collagenase (type XI), 1 mg·mL^−1^ soybean trypsin inhibitor, and 1 mg·mL^−1^ bovine serum albumin, which was gently bubbled with a 95% O_2_–5% CO_2_ gas mixture to gently stir the enzyme solution, as previously described [52]. After isolation, cell suspension was stored in 0.05 mM Ca^2+^ external solution containing 20 mM taurine and 0.5 mg·mL^−1^ bovine serum albumin, at 4 °C under normal atmosphere.

#### 3.4.3. Whole-Cell Patch Clamp Recordings

Cells were continuously superfused with external solution containing 0.1 mM Ca^2+^ and 30 mM TEA using a peristaltic pump (LKB 2132, Bromma, Sweden), at a flow rate of 400 µL·min^−1^. The conventional whole-cell patch-clamp method [53] was employed to voltage-clamp smooth muscle cells. Recording electrodes were pulled from borosilicate glass capillaries (WPI, Berlin, Germany) and fire-polished to obtain a pipette resistance of 2–5 MW when filled with internal solution [containing, in mM: 100 CsCl, 10 Hepes, 11 EGTA, 1 CaCl_2_ (pCa 8.4), 2 MgCl_2_·6 H_2_O, 5 Na-pyruvate, 5 succinic acid, 5 oxaloacetic acid, 3 Na_2_-ATP, and 5 phosphocreatine; pH was adjusted to 7.4 with CsOH]. Ca^2+^ concentration was calculated using the computer programme EqCal (BioSoft, Cambridge, UK) by taking into account pH and Mg^2+ ^concentration, as described by Fabiato & Fabiato [54]. An Axopatch 200B patch-clamp amplifier (Molecular Devices Corporation, Sunnyvale, CA, USA) was used to generate and apply voltage pulses to the clamped cells and record the corresponding membrane currents. At the beginning of each experiment, the junction potential between the pipette and bath solution was electronically adjusted to zero. Current signals, after compensation for whole-cell capacitance and series resistance (between 70%–80%), were low-pass filtered at 1 kHz and digitized at 3 kHz prior to being stored on the computer hard disk. Electrophysiological responses were tested at room temperature (20–22 °C).

#### 3.4.4. I_Ba_ Recordings

Cells used in this study expressed both LTCC and T-type Ca^2+^ channels [55]. I_Ba_ was always recorded in external solution containing 30 mM TEA as well as 5 mM Ba^2+^. Current was elicited with 250-ms clamp pulses (0.067 Hz) to −40 mV or 0 mV from a V_h_ of −80 mV. Data were collected once the current amplitude had been stabilised (usually 7–10 min after the whole-cell configuration had been obtained). Under these conditions I_Ba(L)_ did not run down during the following 40 min [56]. K^+^ currents were blocked with 30 mM TEA in the external solution and Cs^+^ in the internal solution. Current values were corrected for leakage and residual outward currents using 10 µM nifedipine, which completely blocked I_Ca(L)_. The osmolarity of the 30 mM TEA- and 5 mM Ba^2+^-containing external solution (320 mosmol) and that of the internal solution (290 mosmol; [57]) were measured with an osmometer (Osmostat OM 6020, Menarini Diagnostics, Florence, Italy). Following control measurements, each cell was exposed to cumulative concentration of a drug by flushing through the experimental chamber recording solution containing the drug. Compounds **30** and **32**, dissolved directly in DMSO, were diluted at least 1000 times prior to use. The resulting concentrations of DMSO (below 0.1%, v/v) failed to alter the response of the preparations. Acquisition and analysis of data were accomplished by using pClamp 8.2.0.232 and 9.2.1.8 software (Molecular Devices Corporation), respectively, and GraphPad Prism version 5.04 (GraphPad Software Inc., San Diego, CA, USA). Data are reported as mean ± SEM; n is the number of cells analysed (indicated in parentheses), isolated from at least 3 animals.

### 3.5. Binding Studies

#### 3.5.1. Cardiomyocytes Isolation

All animal care and experimental procedures complied with the International guidelines for the Care and Use of Laboratory Animals and were approved by the Animal Care and Ethics Committee of the Università di Siena, Italy. Single cardiac myocytes (CM) were isolated from male Sprague−Dawley rats (Charles River Italia, Como, Italy), injected with 500 U/100 g b.w. heparin i.p., anaesthetized (i.p.) with a mixture of Ketavet^®^ (30 mg·kg^−1^ ketamine; Intervet, Aprilia, Italy) and Xilor^®^ (8 mg·kg^−1^ xylazine; Bio 98, San Lazzaro, Italy), decapitated, and bled. After thoracotomy, the heart was rapidly removed, mounted on a micro-Langendorff apparatus, and perfused for 20 min at 37 °C with a nominally Ca^2+^-free solution (low Ca^2+^ solution, LCS) of the following composition (mM): 120 NaCl, 10 KCl, 10 HEPES, 10 glucose, 1.2 MgCl_2_, 1.2 KH_2_PO_4_, 5 Na-pyruvate, and 20 taurine at pH 7.2, equilibrated with 95% O_2_/5% CO_2_. The solution was then quickly changed to LCS complemented with 0.9 mg/mL of collagenase Type I (Sigma Chimica, Milan, Italy), 0.05 mg/mL of Dispase I (Roche Gmbh, Penzbeg, Germany), and 1.5 mg/mL of acid-free bovine serum albumin (Sigma Chimica, Milan, Italy) for 10 to 15 min. When the heart was soft, perfusion was stopped, and the tissue was chopped into small pieces and gently stirred in fresh LCS at room temperature. The cardiomyocytes that appeared in the supernatant were purified by centrifugation (5 min at 800*g*) and frozen at −80 °C until use. Pooled cells derived from at least three animals have been used for each binding experiment. Protein concentrations were estimated by using the method of Bradford with BSA as the standard.

#### 3.5.2. [^3^H]Diltiazem Binding Assays

Aliquots of defrozen rat CM (200 μg) were incubated with ligands in 50 mM Tris buffer (pH 7.4) at 25 °C for 90 min in a final volume of 0.2 mL. For heterologous competition curves, fixed amounts of the tracer (5 nM) were displaced by increasing concentrations of several unlabeled ligands (0.1 nM−100 μM). Incubation was terminated by rapid filtration on Whatman GF/B glass fiber filters (presoaked for at least 1 h in polyethyleneimmine 0.5%) and washed three times with 3 mL of ice-cold wash buffer. The filters were then placed in scintillation vials, 5 mL of liquid scintillation added, and the radioactivity was determined by liquid scintillation using Perkin Elmer TRI-CARB 1900 TR instrument (Perkin–Elmer Life Science, Boston, MA, USA). Nonspecific binding was defined by means of 100 μM unlabeled diltiazem. All of the experiments were always run in triplicate.

### 3.6. Multidrug Resistance Studies

The potentiation of antiproliferative activity of oxadiazolothiazinones **25**–**27** was evaluated by the MTT test using an oxadiazolothiazinone concentration causing *per se* 5% inhibition of cell proliferation, as described in reference [13].

### 3.7. Molecular Modelling of Calcium Channels Blockers

The version 2.0 of the software FLAP [58] was used to develop all the in silico models described. Several features were used, including: database generation, molecular alignment, 3D-QSAR, pharmacophore generation, and docking of the pharmacophore. Details are reported below.

*Database*: A database of oxadiazolothiazinones was created with the following options: up to 50 conformers; minimum RMSD between two conformers set to 0.1 Å; GRID probes DRY, O and N1.

Alignment. An automatic alignment was obtained for the 29 molecules of the dataset (see Supplementary, Table S1) that are from the present paper and from references [11,12,26]. The procedure selects the two compounds with the highest similarity (based on molecular graphs) and aligns them. Then, the alignment of all the other molecules is run iteratively, with the set of templates increased of one molecule after each run. Among the criteria to build the molecular graphs, atom numbers were used. The alignment consists of the superimposition of the common substructure, followed by a geometrical relaxation, repeated several times in order to achieve an optimal solution (number of conformers set to 10).

*3D-QSAR variables.* The block of the probe O and the blocks for the pseudofields POSITIVE (POS) and NEGATIVE (NEG) were excluded. This was carried out by selecting the option Pretreatment, excluding the mentioned blocks, and assigning weight = 1.0 to the other blocks that were used (probes H, DRY, N1, pseudofields HYD, DON, ACC, AROM, HAL).

3D-QSAR regression method. An algorithm named IVPLS (Iterative Variable Simplification-Partial Least Squares) was used. This is a modified PLS in which a dimension-wide variable simplification is carried out.

*Pharmacophore generation.* The 3D structures of **25**, **28**, **29**, **48** (P1 in reference [36]), **49** (B3 in reference [36]), **50** (M8 in reference [36]), and diltiazem were imported in FLAP as described above (Database), then up to 30 additional conformations were generated on-the-fly by FLAPpharm, that filters the conformations in order to keep the most pharmacophorically similar ones, and then performs a pruned tree search to find common alignment models; this was done with the option “Quick model”. The best model was selected; it is a pseudomolecule with a score of 0.958.

*Docking of the LTCC pharmacophore.* The file with the coordinates of a single snapshot of the homology model of L-type Ca^2+^ channel with diltiazem was received from Prof B. Zhorov [43]. The homology model was imported into FLAP, and the selected pharmacophoric pseudomolecule docked into the channel site using the “pseudo high” option; in other words the atom-centred pseudofields of the pharmacophore were matched to the GRID MIFs of the channel, with the “high strictness” setting. The higher strictness filters the model points and fields to leave those that are common to 60% or more of the aligned input molecules. The pharmacophoric pseudomolecule was docked into the protein and the first solution of the docking analysis by pose, according to the “Global Product Score”, was taken as result of the pharmacophore-docking.

### 3.8. Molecular Modelling of P-glycoprotein Inhibitors

*PGP Docking simulation*. All the protein target structures were built by homology using the software MODELLER [59]. The model utilized as target in our simulations was the NBD2 domain built by homology using the coordinates of the human NBD1 domain (PDB code 2CBZ) as a template [60]. Each moiety was docked to the protein model using the software AutoDock 3.05 with the macromolecule considered as a rigid body and the ligands being flexible. In the case of NBD2 domain the grid was extended over the whole protein. A grid spacing of 0.375 Å was used to build affinity maps for all the atoms present, and an electrostatic map. The correct positioning of the ligands within the active site cleft we validated using a second docking program, GOLD (CCDC, Cambridge, UK) which also allowed us the calculation of a fitness function (Goldscore) in order to confirm our findings obtained by the with AutoDock software

## 4. Conclusions

For this special issue on *in silico drug design and in silico screening* we have reported the case study of oxadiazolothiazinones. We describe their synthesis through a ring-into-ring conversion by applying the Cusmano-Ruccia reaction. When specifically decorated at position-8 with an OR group (with R = alkyl chain) and a substituted phenyl, this scaffold exerts a negative inotropic activity on guinea-pig heart, and inhibits human P-glycoprotein.

In the past, we have studied the ability of this series of oxadiazolothiazinones to decrease the inotropy, developed a QSAR model, and used the most potent oxadiazolothiazinone as a template for ligand-based virtual screening. Here, we enlarge the chemical decorations of the oxadiazolothiazinone scaffold, and present for the first time the synthesis and* in vitro* data for 11 new derivatives. According to data for guinea-pig isolated left and right atria, we found two compounds with negative inotropic activity (with submicromolar potency) and two with negative chronotropic activity. We also tested all the molecules on vascular and nonvascular smooth muscle: every compound except **30** and **34** is inactive in relaxing vascular smooth muscle. As regards the activity relaxing the nonvascular smooth muscle, all compounds except **25** were slightly active.

We investigated two compounds, **30** and **32**, in more detail, under experimental conditions that allow for the identification of blockers of LTCC. High K^+^-induced contraction of aorta rings, in fact, is the result of an increased Ca^2+^ influx through LTCC and is specifically inhibited by Ca^2+^-antagonists. Both compounds antagonize high K^+^-induced contraction in a concentration-dependent manner, interpretable as a consequence of the blockade of LTCC. We also studied the binding at the benzothiazepine-binding site: the complex interaction exerted by **32** might reflect a positive allosteric modulation at the diltiazem binding site, as already observed for **25** [36].

With the functional data we have developed a new 3D-QSAR model for negative inotropic potency: with very recent in silico techniques, we have built a quantitative in silico model which will be used to *design* further derivatives, and obtained a pharmacophore that may be useful for *screening*. The new model is currently in use for the prediction of the activity of further derivatives.

A key role in the biologically relevant interactions, observed with different protein systems (ion channel and P-glycoprotein), is played by the oxadiazolone moiety: the distribution of the molecular electrostatic potential has shown that the two oxygen atoms generate a region of significant interaction with a positive charge. In the case of ion channels, given the presence of the positively charged calcium ions in the transmembrane protein LTCC, we hypothesize the formation of a ternary complex between the molecule (oxadiazolothiazinone), the ion (Ca^2+^) and the protein (LTCC): we elaborated and verified this hypothesis by means of pharmacophore generation (based on a set of diverse molecules that we identified as active against LTCC) and through the docking of the pharmacophore into a homology model of the protein. In the case of P-glycoprotein, given the presence of positively charged residues (Arg) in the binding pocket, we hypothesize a strong interaction of the oxadiazolothiazinone moiety: we supported this hypothesis by calculating the Molecular Electrostatic Potential and observing a significant complementarity between the two partners, oxadiazolothiazinone and P-glycoprotein.

## Supplementary Materials

Supplementary materials can be accessed at: http://www.mdpi.com/1420-3049/19/10/16543/s1.

## Supplementary Files

Supplementary File 1
