# Supplementary material for: Playing with Opening and Closing of Heterocycles: Using the Cusmano-Ruccia Reaction to Develop a Novel Class of Oxadiazolothiazinones, Active as Calcium Channel Modulators and P-Glycoprotein Inhibitors
Source: Molecules. 2014 Oct 14;19(10):16543–72. doi: 10.3390/molecules191016543 (PMC6271282; doi:10.3390/molecules191016543)
Supplement: Supplementary File 1 [file molecules-19-16543-s001.pdf]

Supplementary Materials

Table S1. Structures and negative inotropic activity data of the 29 compounds of the training set.

Source [a]	Molecule	Structure [b]	EC_{50} (μM)	pEC_{50}
[1]	**4b**	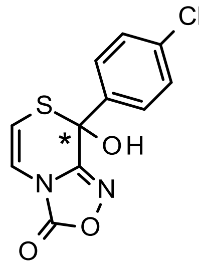	4.07	5.390
[1]	**4c**	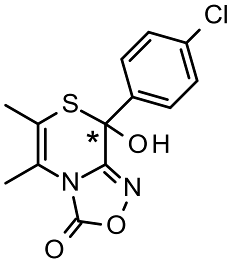	1.42	5.848
[1]	**4i**	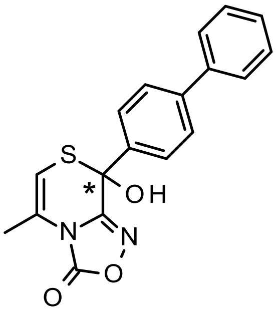	2.65	5.577
[1]	**4l**	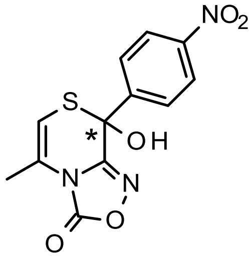	1.65	5.783
[1]	**4n**	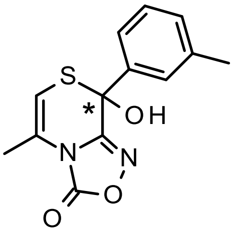	0.65	6.187
[1]	**4p**	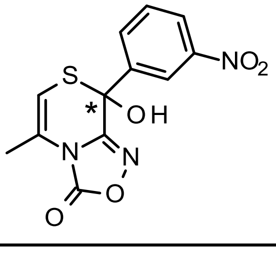	1.76	5.754

Table S1. *Cont.*

Source [a]	Molecule	Structure [b]	EC_{50} (μM)	pEC_{50}
[1]	**4q**	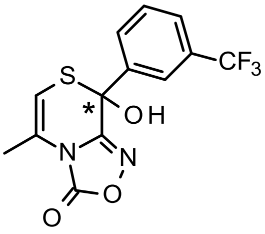	2.17	5.664
[1]	**6**	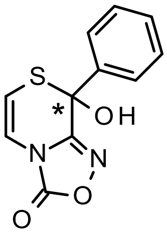	6.58	5.182
[2]	**1a**	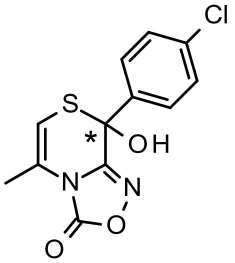	0.80	6.097
[2]	**1b**	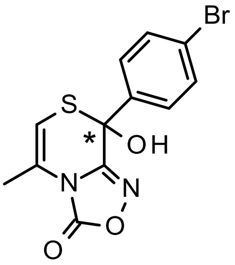	0.32	6.495
[2]	**5a**	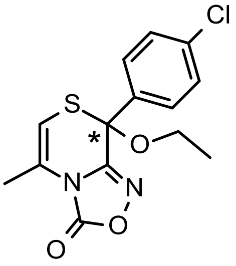	0.27	6.569
[2]	**14a**	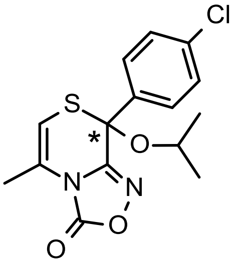	0.22	6.658

Table S1. *Cont.*

Source [a]	Molecule	Structure [b]	EC_{50} (μM)	pEC_{50}
[2]	**20a**	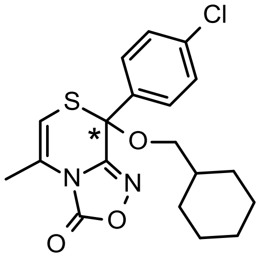	0.24	6.620
[2]	**20b**	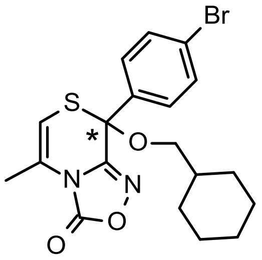	0.19	6.721
[2]<br>Present paper	**5b**<br>**25**	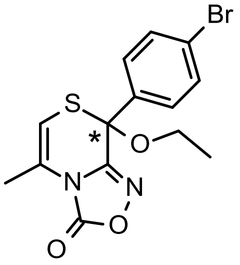	0.04	7.398
[3]<br>Present paper	**2b**<br>**26**	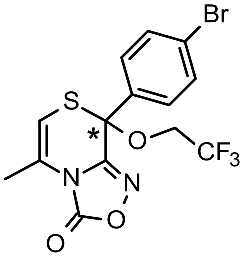	0.022	7.658
[3]<br>Present paper	**3a**<br>**28**	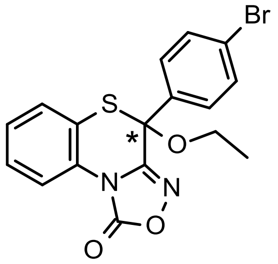	0.013	7.886
[3]<br>Present paper	**3b**<br>**29**	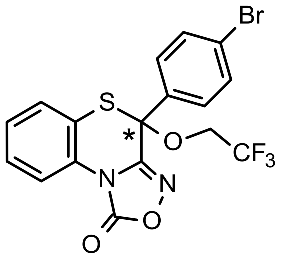	0.006	8.222

Table S1. *Cont.*

Source [a]	Molecule	Structure [b]	EC_{50} (μM)	pEC_{50}
Present paper	**30**	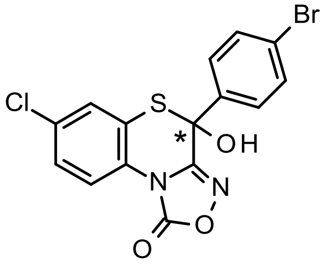	0.36	6.444
Present paper	**31**	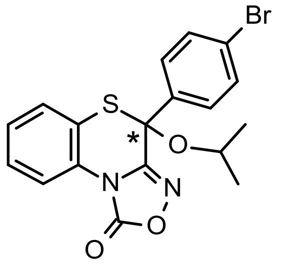	0.44	6.357
Present paper	**32**	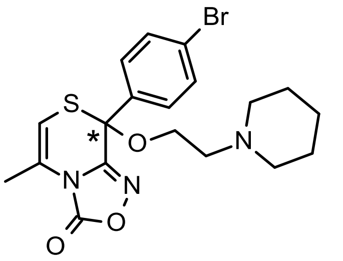	3.67	5.435
Present paper	**33**	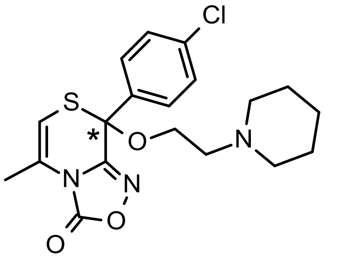	5.25	5.280
Present paper	**34**	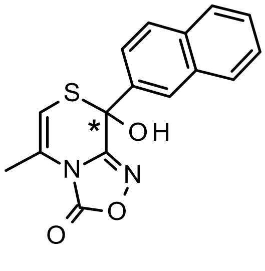	0.057	7.244
Present paper	**35**	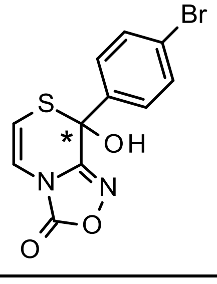	0.13	6.886

Table S1. *Cont.*

Source [a]	Molecule	Structure [b]	EC_{50} (μM)	pEC_{50}
Present paper	**36**	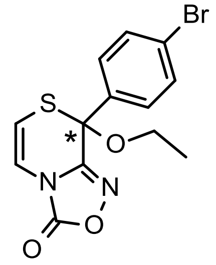	0.039	7.409
Present paper	**37**	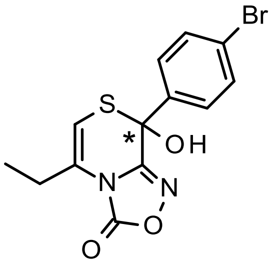	1.11	5.955
Present paper	**38**	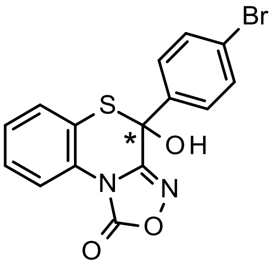	4.23	5.374
Present paper	**39**	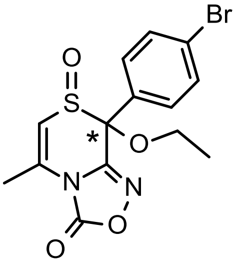	1.08	5.967
Present paper	**40**	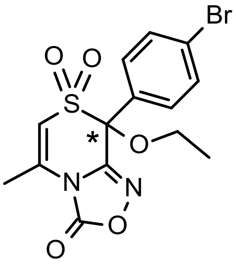	0.76	6.119

[a] References are listed below. [b] Although the data refer to the racemic mixture of the compounds, we used in the modeling the *R*-form.

References

1. Budriesi, R.; Cosimelli, B.; Ioan, P.; Lanza, C.Z.; Spinelli, D.; Chiarini, A. Cardiovascular characterization of [1,4]thiazino[3,4-*c*][1,2,4]oxadiazol-3-one-derivatives: Selective myocardial calcium channel modulators. *J. Med. Chem.* **2002**, *45*, 3475–3481.
2. Budriesi, R.; Carosati, E.; Chiarini, A.; Cosimelli, B.; Cruciani, G.; Ioan, P.; Spinelli, D.; Spisani, R. A New Class of Selective Myocardial Calcium Channel Modulators. 2. The Role of the Acetal Chain in Oxadiazol-3-one Derivatives. *J. Med. Chem.* **2005**, *48*, 2445–2456.
3. Carosati, E.; Ioan, P.; Barrano, G.B.; Caccamese, S.; Cosimelli, B.; Devlin, F.J.; Severi, E.; Spinelli, D.; Superchi, S.; Budriesi, R. Synthesis and Absolute Configuration Determination of New Oxadiazolothiazinones Active as L-Type Calcium Channel Blockers. *Eur. J. Med. Chem.* **2014**, submitted.
